# Relationship between practices of eye protection against solar ultraviolet radiation and cataract in a rural area

**DOI:** 10.1371/journal.pone.0255136

**Published:** 2021-07-29

**Authors:** Li-Ju Chen, Yun-Jau Chang, Chun-Fu Shieh, Jy-Haw Yu, Ming-Chin Yang

**Affiliations:** 1 Department of Ophthalmology, Heping Fuyou Branch, Taipei City Hospital, Taipei, Taiwan; 2 University of Taipei, Taipei, Taiwan; 3 Institute of Health Policy and Management, College of Public Health, National Taiwan University, Taipei, Taiwan; 4 Department of Surgery, National Taiwan University Hospital, Taipei, Taiwan; 5 Department of Surgery, Zhongxing branch, Taipei City Hospital, Taipei, Taiwan; 6 Lienchiang County Hospital, Matsu, Taiwan; National Eye Institute, UNITED STATES

## Abstract

**Background:**

Cataract is a public health concern worldwide that differentially affects rural residents of outlying islands where ultraviolet radiation (UVR) may have greater penetration because of less shading.

**Objectives:**

To assess the relationships between attitudes and practices of eye protection and eye diseases for residents of an offshore island of Taiwan.

**Methods:**

Questionnaire survey was administered to local residents (age > 50 years) regarding socio-demographic information, attitudes/practices of eye protection under sun exposure and eye diseases.

**Results:**

A total of 816 participants (response rate 90.7%, 816/900) completed the questionnaires. Mean age was 63.7 (+ 10.8) years. Among these participants, 44.4%, 15.1% and 8.3% had cataract, dry eye and glaucoma, respectively. Although 86.3% and 88.2% of participants agreed that they should avoid outdoor activities and wear glasses/broad-brimmed hats in harsh daylight, 69.4% and 48.3% of participants never/rarely used glasses or hats/umbrellas in harsh daylight, respectively. Predictors of less practices of eye protection against solar UVR included residents who were male, with lower education level, with longer residence and lack of commercial health insurance. Multivariate logistic regression revealed that practices of eye protection under sun exposure were significantly associated with less cataract, but not glaucoma or dry eye. Participants who did not wear glasses, broad-brimmed hats/use umbrellas or both in harsh sunlight (almost) every time were respectively associated with a 57% (*P* = 0.028), 45% (*P* = 0.027) or 70% (*P* = 0.026) increase of cataract than those who did in harsh sunlight (almost) every time.

**Conclusions:**

Practices of eye protection under sun exposure is associated with lower risk of cataract.

## Introduction

Cataract, primarily an age-related disorder, is the leading cause of visual impairment all over the world (except developed countries) [1, 2]. Studies have supported the role of high cumulative ultraviolet radiation (UVR) exposure in development of cortical [3, 4], nuclear and posterior subcapsular cataracts [5]. Via appropriate ultraviolet protection, the cataract burden could be reduced by roughly 5% or even higher [6]. Although damaging effects of excess solar UVR exposure on the skin have been well-known by medical university students (about 95%), disproportionate students (27.8%) recognized the eye damage such as cataract could result from UVR [7]. The attitude toward eye protection is frequently ignored and specific guidance is not emphasized when sunglasses are recommended [8].

Simple behavioral changes, appropriate clothing, shade use (wearing hats or using umbrellas), and use of UVR blocking spectacles, sunglasses or contact lenses are effective measures for ocular protection against UVR [9, 10]. The effectiveness depends upon the percentage of transmission blockage of diffuse direction of UVR and its reflection of the irradiance over eyes. Shade seeking may prevent possible ocular side effects of UVR only when a person faces the shaded areas [11]. Even when facing away from the sun, the eyes are subject to ground reflection [12]. A beach umbrella could intercept all direct UVR but approximately 34% of the incident horizontal irradiance was not intercepted by the umbrella [13]. The use of brimmed-baseball caps, broad-brimmed hats or spectacles might offer a 22–95%, 50% or 62–94% reduction of UV exposure to the eyes, respectively [14, 15]. Sunglasses, clear lenses (plano and prescription) and some contact lenses reduce UVR markedly, however, back reflectance property of the lens and frame characteristics vary and thus influence the effect of UVR protection [12]. Calculating exposure to sunlight, some investigators designated ocular UVR cut-off rate as 0.47 for hats/umbrella and 0.79 for sunglasses [16], while another study used UVR cut-off rate as 0.2 for hats and 0.9 for glasses/sunglasses [5].

Rural residents are exposed to higher levels of UVR than their urban counterparts because of less shading, excessive exposure is directly associated with eye diseases [17]. Disability and medical expenditure owing to visual impairment may aggravate the disease burden of rural areas. Taiwan consists of a number of sparsely populated mountainous areas and remote islands where solar UVR is harsh and buildings might not provide ideal sunlight shade. Health promoting campaigns including sun protection against eye diseases were frequently held, residents of these areas still had high prevalence of eye diseases [18]. Little is known about the overall use rate of sunglasses in rural areas of Taiwan, especially in outlying island’s where UVR is harsh.

This study aimed to investigate the attitudes/practices of eye protection against solar UVR, eye diseases and their associations in residents of an offshore island of Taiwan on a community basis. We also explored the predictors of attitudes/practices to help identify groups with lower compliance.

## Materials and methods

Between May and June 2014, residents (aged 50 and older) in Nangan (major island of Matsu) were invited to participate in this questionnaire survey. Matsu Archipelago, a renowned subtropical military stronghold and historic front of Taiwan, lies about 200 kilometers off Taiwan proper by Taiwan Strait. Residents of Nangan accounted for more than 60% population of Matsu, according to national census [19]. During this period annually, local health authority aligns with the Health Promotion Administration launch of a community-based integrated screening project [including slit lamp biomicroscopy (for ocular surface and cataract) and eye fundus examination (for optic disc)] for residents of Matsu. Residents who received annual health screening were well informed about their ophthalmic diseases. The accuracy of ophthalmic conditions of participants after this examination was considered dependable and appropriate for conducting of a survey regarding common eye diseases. Criterion of age selection was set on the fact that two-thirds of visual impairment (including blindness) worldwide occurred in this age group [20]. Participants in this survey were required to have sufficient cognitive capability to comprehend and finish the interviewer-administered person-to-person inquiry. Experienced interviewers were selected from local residents and they needed to attend seminars assembled by one of the authors in advance to expedite the survey. The constructed questionnaire (S1 and S2 Files) contained two sections (the first section included questions related to clinical information and eye diseases; the second section included questions related to health habits, daily sunlight exposure as well as attitudes and practices of eye protection under harsh sunlight).

Attitudes of eye protection included two items that participants were asked to respond whether they agreed/disagreed they should avoid outdoor activities in harsh sunlight or should wear a broad-brimmed hat or eyeglasses (including clear prescription lenses, contact lenses, sunglasses) in harsh sunlight. Practices of eye protection (in recent one year) included five items: did they work or exercise in windy outdoor space every day, did they work or exercise beside transparent windows in the daytime every day, did they work or exercise in harsh sunlight every day, did they wear glasses (including clear prescription lenses, contact lenses, sunglasses) in harsh sunlight every time, did they wear a broad-brimmed hat or use an umbrella in harsh sunlight every time? Information regarding socio-demographic data included age, gender, residential village, years of residence, level of highest education, occupation, marital status, religion, annual household income and commercial health insurance (a surrogate for socio-economic status). The questionnaire was inspected thoroughly and carefully by five experts for content validity. All of the participants responded these questionnaires anonymously. The institutional review board of Taipei City Hospital approved this survey (reference number: TCHIRB-1020122-E) and waived signed consent sheet. The survey was performed in compliance with the guidelines of the Declaration of Helsinki.

Dependent variables were common eye diseases among participants. Categorical variables between practices of eye protection under sun exposure and attitudes of ocular photoprotection were analyzed using the *X*^2^ test. Logistic regression was used to analyze relationship between practices of eye protection under sun exposure and eye diseases (dry eyes, cataract and glaucoma). Predictors of practices were analyzed using multivariate logistic regression. Odds ratio (OR) with 95% confidence interval (CI) were reported. A *P* < 0.05 (two-tailed) was considered as statistically significant.

## Results

A total of 851 questionnaires were obtained after 900 questionnaires were administered via interviewers during the survey period. Thirty-five questionnaires did not provide sufficient information for this study and were thus excluded. Finally, 816 questionnaires were deemed as adequate and eligible for this study (response rate 90.7%). Enrolled participants represented 63% of the total inhabitants in Matsu (Nangan) of the study age group. Basic socio-demographic characteristics, health habits (alcohol consumption and smoking) and known diseases of enrolled participants are summarized in Table 1. The average age of participants was 63.7 years (range 50–100). Females comprised of 51.7% of the participants and predominated in each age group. Most participants (75.7%) had resided in Matsu for more than 40 years and nearly a half of the participants (50.1%) resided in the vicinity of healthcare service providers. Education level of senior high school or higher accounted for 33.0% and illiteracy accounted for 23.7% of the participants. Fifty-six percent of the participants belonged to working group. Only 45.3% of the participants were covered by commercial health insurance. More than half of the participants had annual household income lower than five hundred thousand New Taiwan dollars (country median: eight point three hundred thousand New Taiwan dollars). Participants reported consuming no alcoholic beverages (56.7%) or tobacco (83.0%), respectively. Three most frequent comorbid conditions were hypertension (52.1%), arthritis (19.2%) and diabetes mellitus (11.8%). Three most frequent eye diseases were cataract (44.4%), dry eye (15.1%) and glaucoma (8.3%).

**Table 1:** Socio-demographic characteristics, health habits, and illness characteristics of study participants (N = 816).

Variable		Number	%
Sex			
	male	394	48.3
	female	422	51.7
Age (years)			
	63.7 (50–100)	+ 10.8	
Residential village			
	Neighborhood of healthcare facility	409	50.1
	Not neighborhood of healthcare facility	407	49.9
Years of residence			
	< 40 years	198	24.3
	> 40 years	618	75.7
Education			
	Below primary school	269	33.0
	Primary school / Junior high school	278	34.1
	Senior high school or higher	269	33.0
Marital status			
	Never married	15	1.8
	Married or cohabiting	644	78.9
	Divorced or separated	25	3.1
	Widowed	109	13.4
	Unspecified	23	2.8
Religion			
	Buddhist/Taoist	738	90.4
	Non-Buddhist/Taoist	78	9.6
Main occupation			
	Working group	459	56.3
	Non-working group	357	43.8
Commercial health insurance			
	Yes	370	45.3
	No	446	54.7
Annual household income*			
	< NTD 500,000	427	52.3
	> NTD 500,000	389	47.7
Alcohol consumption			
	No	463	56.7
	Yes	353	43.3
Tobacco use			
	No	677	83.0
	Yes	139	17.0
Comorbid condition (multiple)^#^			
	Hypertension	425	52.1
	Rheumatoid arthritis	157	19.2
	Diabetes	96	11.8
Eye diseases diagnosed by ophthalmologist (multiple)^#^			
	Cataract	362	44.4
	*Cataract (mild)*	*248*	*30*.*4*
	*Cataract (moderate or severe)*	*114*	*14*.*0*
	Dry eye	123	15.1
	Glaucoma	68	8.3
	Retinal disease	40	4.9
	High myopia (< -6 Diopters)	34	4.2
	Total	816	100

NTD: New Taiwan dollar

*median (country): eight point three hundred thousand New Taiwan dollars

^#^indicate numbers would not sum up as 816.

The most frequently reported daily sun exposure duration was less than three hours (74.5%, see Table 2). Regarding attitudes of eye protection under sun exposure, 86.3% of participants agreed that outdoor activity in harsh sunlight should be avoided and 88.2% of participants agreed that broad-brimmed hats or eyeglasses (including contact lenses, sunglasses) should be worn in harsh sunlight. Regarding practices of eye protection under sun exposure, only 20.0% of participants worked or exercised in windy outdoor spaces almost every day, 21.7% of participants worked or exercised beside transparent windows in the daytime almost every day, 20.3% of participants worked or exercised in harsh sunlight almost every day, 19.6% of participants wore eyeglasses (including contact lenses, sunglasses) in harsh sunlight almost every time and 30.8% of participants wore broad-brimmed hats or used umbrellas in harsh sunlight almost every time. There were 69.4% and 48.3% of participants who have never/rarely used eyeglasses or never/rarely used broad-brimmed hats/ umbrellas in harsh sunlight, respectively. Attitudes of sun protection (agreed to avoid outdoor activities or wear broad-brimmed hats/glasses) in harsh sunlight were associated with frequent use of broad-brimmed hats/umbrellas or glasses, while they were not associated with frequent working/exercising in harsh sunlight (Table 3).

**Table 2:** Attitudes of eye protection under sun exposure and pattern of daily practices.

Variable		Number	%
**Daily sun exposure time (hours)**			
	< 3	608	74.5
	3 ~ 5	108	13.2
	> 5	100	12.3
**Attitude of eye protection under sun exposure**			
* Avoid outdoor activities in harsh sunlight*			
	Agree	704	86.3
	Indifferent	85	10.4
	Disagree	27	3.3
* Wear a broad-brimmed hat or eyeglasses (including clear prescription lenses*, *contact lenses*, *sunglasses) in harsh sunlight*			
	Agree	720	88.2
	Indifferent	84	10.3
	Disagree	12	1.5
**Practice of eye protection under sun exposure**			
* Work or exercise in windy outdoor space*			
	Every day/almost every day	163	20.0
	Not every day	653	80.0
* Work or exercise beside transparent windows in the daytime*			
	Every day/almost every day	177	21.7
	Not every day	639	78.3
* Work or exercise in harsh sunlight*			
	Every day/almost every day	166	20.3
	Not every day	650	79.7
* Wear eyeglasses (including clear prescription lenses*, *contact lenses*, *sunglasses) in harsh sunlight*			
	Every time/almost every time	160	19.6
	Often/sometimes/rarely	355	43.5
	Never	301	36.9
* Wear a broad-brimmed hat or use an umbrella in harsh sunlight*			
	Every time/almost every time	251	30.8
	Often/sometimes/rarely	440	53.9
	Never	125	15.3
* Simultaneously use glasses and a broad-brimmed hat/an umbrella in harsh sunlight*			
	Every time/almost every time	113	13.8
Total		816	100.0

**Table 3:** Relationship between attitudes and practices of eye protection under sun exposure.

Attitude	Practice	OR	(95% C.I.)	*P* value
*Avoid outdoor activities in harsh sunlight* (disagree or indifferent)	*Frequently work or exercise in windy outdoor space*	1.16	(0.75–1.82)	0.501
	*Frequently work or exercise beside transparent windows in the daytime*	1.31	(0.84–2.02)	0.238
	*Frequently work or exercise in harsh sunlight*	0.96	(0.63–1.47)	0.850
	*Frequently wear glasses in harsh sunlight*	3.53	(1.97–6.31)	< 0.001
	*Frequently wear a broad-brimmed hat or use an umbrella in harsh sunlight*	2.04	(1.35–3.08)	0.001
*Wear a broad-brimmed hat or eyeglasses in harsh sunlight* (disagree or indifferent)	*Frequently work or exercise in windy outdoor space*	0.91	(0.57–1.43)	0.667
	*Frequently work or exercise beside transparent windows in the daytime*	1.01	(0.64–1.58)	0.978
	*Frequently work or exercise in harsh sunlight*	0.81	(0.52–1.27)	0.363
	*Frequently wear glasses in harsh sunlight*	4.30	(2.19–8.43)	< 0.001
	*Frequently wear a broad-brimmed hat or use an umbrella in harsh sunlight*	2.24	(1.44–3.50)	< 0.001

Characters in parentheses indicate reference groups; OR: odds ratio (Chi-square test); C.I.: confidence interval; ‘*frequently’* indicates every day/almost every day or every time/almost every time.

Table 4 summarizes the relationships between practices of eye protection under sun exposure and individual common eye diseases. There was no significant association between any practices of eye protection under sun exposure and dry eye disease. Neither was there significant association between any practices of eye protection under sun exposure and glaucoma. However, there were significant associations between practices of eye protection under sun exposure and cataract. Participants who did not wear glasses in harsh sunlight every time (or almost every time) had higher risk of cataract (OR 1.57, 95% CI 1.05–2.34, *P* = 0.028) than those who did so every time (or almost every time). Similarly, participants who did not wear broad-brimmed hats or use umbrellas in harsh sunlight every time (or almost every time) had higher risk of cataract (OR 1.45, 95% CI 1.04–2.02, *P* = 0.027) than those who did so in harsh sunlight every time (or almost every time). Compared to those who simultaneously used glasses and hats/umbrellas in harsh sunlight, participants who neither used glasses nor used hats/umbrellas in harsh sunlight every time (or almost every time) were associated with higher risk of cataract (OR 1.70, 95% CI 1.07–2.70, *P* = 0.026). Predictors of participants who did not wear glasses in harsh sunlight (almost) every time were education and commercial health insurance (Table 5). Participants with lower education level (< junior high school) and lack of commercial health insurance tended not to wear glasses in harsh sunlight (almost) every time (OR 1.91, 95% CI 1.19–3.05, *P* = 0.007; OR 1.90, 95% CI 1.25–2.91, *P* = 0.003, respectively). Predictors of participants who did not wear broad-brimmed hats or use umbrellas in harsh sunlight (almost) every time were sex and years of residence. Participants with male gender and longer residence (greater than 40 years) tended not to wear broad-brimmed hats or use umbrellas in harsh sunlight (almost) every time (OR 1.42, 95% CI 1.02–1.96, *P* = 0.038; OR 1.71, 95% CI 1.19–2.47, *P* = 0.004, respectively).

**Table 4:** Relationship between practices of eye protection under sun exposure and eye diseases.

		Dry eye			Cataract			Glaucoma	
Practices of eye protection and daily pattern	OR*	(95% C.I.)	*P* value	OR*	(95% C.I.)	*P* value	OR*	(95% C.I.)	*P* value
*Work or exercise in windy outdoor space (almost every day)*	1.57	(0.88–2.79)	0.126	1.20	(0.81–1.80)	0.366	1.62	(0.75–3.51)	0.222
*Work or exercise beside transparent windows in the daytime (almost every day)*	1.41	(0.89–2.20)	0.171	1.20	(0.82–1.75)	0.345	1.46	(0.73–2.94)	0.286
*Work or exercise in harsh sunlight (almost every day)*	1.64	(0.92–2.93)	0.095	1.12	(0.75–1.68)	0.578	1.23	(0.59–2.54)	0.580
*Wear glasses in harsh sunlight (almost every time)*	0.81	(0.50–1.30)	0.373	1.57	(1.05–2.34)	0.028	0.74	(0.40–1.37)	0.340
*Wear a broad-brimmed hat or use an umbrella in harsh sunlight (almost every time)*	0.77	(0.51–1.15)	0.202	1.45	(1.04–2.02)	0.027	1.25	(0.71–2.20)	0.443
*Simultaneously use glasses and a broad-brimmed hat/an umbrella in harsh sunlight (almost every time)*	0.85	(0.50–1.46)	0.560	1.70	(1.07–2.70)	0.026	2.11	(0.72–5.41)	0.122

Characters in parentheses indicate reference groups; OR: odds ratio (Chi-square test); C.I.: confidence interval

*: estimates were adjusted for age, sex, residential village, duration of residence, education level, income status and daily sun exposure time; almost every day or almost every time includes every day or every time.

**Table 5:** Relationship between socio-demographic characteristics and practices of eye protection under sun exposure.

	*Wearing eye glasses in harsh sunlight*, *almost every time*			*Wearing a broad-brimmed hat or using an umbrella in harsh sunlight*, *almost every time*		
Variables	OR	(95% C.I.)	*P* value	OR	(95% C.I.)	*P* value
Sex (male)	1.30	(0.88–1.93)	0.188	1.42	(1.02–1.96)	0.038
Age (> 60 years)	1.38	(0.84–2.28)	0.207	0.87	(0.57–1.31)	0.495
Village (not neighborhood of healthcare facility)	0.89	(0.62–1.29)	0.545	0.84	(0.62–1.15)	0.275
Years of residence (> 40)	1.31	(0.86–1.98)	0.207	1.71	(1.19–2.47)	0.004
Education (≤ junior high school)	1.91	(1.19–3.05)	0.007	0.78	(0.52–1.19)	0.251
Marital status (not married/cohabiting)	1.37	(0.84–2.23)	0.211	1.35	(0.92–2.00)	0.125
Religion (non-Buddhist or Taoist)	0.92	(0.52–1.65)	0.787	1.32	(0.77–2.26)	0.316
Occupation (not working group)	0.98	(0.62–1.57)	0.942	1.34	(0.92–1.96)	0.123
Commercial health insurance (no)	1.90	(1.25–2.91)	0.003	1.11	(0.78–1.58)	0.578
Annual household income (≤ NTD 500,000)	1.26	(0.84–1.88)	0.261	1.38	(0.99–1.92)	0.059

OR: odds ratio; C.I.: confidence interval; NTD: New Taiwan dollar; characters in parentheses indicate reference groups; almost every day or almost every time includes every day or every time.

## Discussion

The current study demonstrated attitudes and practices of ocular protection against harsh sunlight and their relationships with common eye diseases for residents of an outlying island of Taiwan. It showed almost ninety percent of participants agreed with the need for ocular protection by avoiding outdoor activities or wearing hats/glasses under harsh sunlight. However, regarding actual practices under harsh sunlight, 69.4% and 48.3% of participants never/rarely used glasses or hats/umbrellas, respectively. No significant associations between attitudes and common eye diseases were found in the current study. Among practices of eye protection under sun exposure, there were two practices that had significant relationships with residents with cataract. Participants who did not wear glasses or wear broad-brimmed hats/use umbrellas in strong sunlight (almost) every time were associated with a 57% and 45% increase of cataract than those who did, respectively. Participants with less education level or no commercial health insurance were less likely to wear glasses in strong sunlight (almost) every time. Participants who lived in Matsu for more than 40 years were less likely to wear broad-brimmed hats/use umbrellas in strong sunlight (almost) every time.

There was less population-based research on the prevalence of cataract in Taiwan reported. A contemporary study of general population aged 40 years and older in Taiwan proper revealed the prevalence of cataract told by medical professionals (excluding mild cataract) was 11.8% [21]. Prevalence of moderate/severe cataract in Matsu (14.0%) was slightly higher than their result as we investigated residents at age 50 years and older. Another study of a specific highly urbanized precinct of Taiwan found the prevalence of cataract for residents aged 50 years and older was 51.0% [22]. Comparing to their data, the total prevalence rate of cataract (44.4%) of our study was lower, which could be due to difference of degree of urbanization. Previous study has shown that the risk of cataract was higher for residents of highly urbanized area [21].

Our findings that a 57%, 45% and 70% reduction of risk of cataract in participants who used glasses, broad-brimmed hats/umbrellas, or both, respectively were paralleled with a study of Taylor et al. showing people who wore hats and sunglasses to protect their eyes from strong sun shine could substantially reduce the risk of cataracts [3]. We confirmed ocular protection against sunlight might decrease risk of cataract formation and our results corresponded with prior suggestion that additional protection means such as broad-brimmed hat might have additive protective effect of UVR when combined with glasses [23]. The current study also could be comparable with the French study, which reported professional exposure to sunlight might increase 63% of posterior subcapsular cataract, but frequent use of sunglasses might offer a 40% decrease in the risk of posterior subcapsular cataract [24]. Based on such evidence, encouraging the public to use hats, sunglasses and umbrellas during period of high UVR is therefore beneficial, effective, and imperative.

Interestingly, knowledge and attitudes toward sunlight protection did not necessarily translate into behavior of practices of ocular protection under harsh daylight as noticed in the current study. Although sunglasses can be used to protect eyes from UVR to reduce risk of cataract, the actual percentage of individuals to wear sunglasses for sunlight protection might be suboptimal. In Hawaii, direct observation in public outdoor recreation settings revealed that only one third of the population wore sunglasses [10]. Cheng et al. observed less than half of the participants in North China used sunglasses (45%) or hats (42%) and nearly half of the participants used sun umbrellas as a protective behavior regarding sun exposure [25].

There was gender difference in practices of ocular protection against solar UVR. Females tended to more frequently wear broad-brimmed hats/use umbrellas under harsh sunlight than males, but there was an insignificant difference between males and females regarding use of glasses in the current study. Gao et al. reported nearly half of female medical university students in northeast China often or always used sun umbrellas as a protection method, which could be considered a preferred and unique approach for sun protection among Chinese women [7]. In a study of Australia, females were prone to frequently wear sunglasses than males [26]. However, in Hawaii, gender was insignificantly associated with the use of sunglasses [10]. Studies are scarce regarding relationship between low education level and ocular photo-protective practices, though association between less education/low income and cataract in Chinese had been reported [27]. For sunscreen use to prevent skin cancer, individuals with low educational level were shown to use less frequently than those with higher educational level [28]. The current study found limited education level and lack of commercial health insurance as predictors of less use of glasses in harsh sunlight.

Outdoor activity increases the potential of long-term solar UVR exposure, especially for those who need to work under harsh light. This in turn may have cumulative impact on formation of cataract. A recent systemic review of 15 studies showed positive association between cataract (cortical cataract most, followed by nuclear cataract) and outdoor work in 12 studies [29]. However, the quality of the analysis performed in that systemic review seemed rather inhomogeneous. Another study estimated risk of cortical cataract in a rural area of east China reported that the risk increased 4.3% when outdoor activity time increased every one hour [4]. However, our results did not support significant association between outdoor activities and cataract. Likewise, studies in rural Myanmar and Mediterranean also reported no significant association between cataract and outdoor occupation [30, 31].

We acknowledge several limitations in the current study. First, the attitudes and practices of eye protection were evaluated under harsh sunlight in the current study which was different from a prior study of 10 am through 2 pm [32]. Though ambient UVR peaks around noon, UVR reaching the eye depends mainly on solar angle [12]. Considering that the geographic characteristics and buildings of Matsu might not provide sufficient shade, harsh sunlight occurred beyond 2 pm, even to 6 pm in summer time. Restricting sunlight exposure time might not reflect the real situation in Matsu. Second, approximately 40% of the residents of Matsu did not attend the survey, as during the study period, many residents were employed in Taiwan or in Mainland China. Third, as this survey was a cross-sectional study, results only indicated the association between ocular protective practices against sunlight and cataract, rather than causal relationships. As such, the results should be interpreted carefully.

## Conclusions

In summary, this study investigated ocular protective attitudes and practices under harsh sunlight and their relationships with common eye diseases for residents of an outlying island of Taiwan. Most participants exhibited appropriate attitudes toward ocular protection under harsh sunlight but this did not convert into actual daily practices in sunny days. About half of participants would not or rarely wear hats/use umbrellas and about 70% of participants would not or rarely wear glasses in harsh sunlight. Regarding common eye diseases of residents, we found a significant association between practices against solar UVR and cataract. Participants who did not wear glasses or wear broad-brimmed hats/use umbrellas in strong sunlight (almost) every time were associated with about a 50% increase of cataract. To reduce barriers, enhanced education may be targeted for residents who are male, with longer duration of residence, limited education level or with no commercial health insurance. Future research may extend to other remote areas to investigate the relationships between attitudes and practices against sunlight exposure and eye diseases.

## Supporting information

S1 File Questionnaire used in the study. English version.
(DOC)

S2 File Questionnaire used in the study. Chinese version.
(DOC)

S3 File Minimal dataset used in the study. (XLS)
