# Supplementary material for: Relationship between practices of eye protection against solar ultraviolet radiation and cataract in a rural area
Source: PLoS One. 2021 Jul 29;16(7):e0255136. doi: 10.1371/journal.pone.0255136 (PMC8321156; doi:10.1371/journal.pone.0255136)
Supplement: S1 File — English version. (DOC) [file pone.0255136.s001.doc]

**Individual health condition**

**1. Do you have the following chronic diseases? (multiple choice)**

| □Nil | □Diabetes | □Hypertension | □CVA |
| --- | --- | --- | --- |
| □Heart disease | □COPD | □Asthma | □Liver cirrhosis |
| □ESRD(HD) | □RA | □Other disease __________ | |

Abbreviations: CVA: cerebrovascular accident; COPD: chronic obstructive pulmonary disease; ESRD: end-stage renal disease; HD: hemodialysis; RA: rheumatoid arthritis

2. Do you need to wear glasses in your daily life (including contact lenses)?

□Yes, please proceed to question 2-1; □No, please skip to question 3

2-1 IF YES: What is the cause that you need to wear glasses? (multiple choice)

□Myopia □Hyperopia □Astigmatism □Presbyopia □Unknown

□Other ___________

3. Have you ever been diagnosed with the following eye diseases by doctors? (Multiple choice)

| □Nil  □Dry eye | □High myopia (refractive error < -6 diopters, either eye)  □Pterygium (flesh growth of the conjunctiva) | | |
| --- | --- | --- | --- |
| □Cataract, mild | □Cataract, moderate | □Cataract, severe | □Glaucoma |
| □Keratopathy | □AMD | □Diabetic retinopathy | □Retinal break/detachment |
| □Ocular trauma | □Eyelid skin cancer | □Trachoma | □Other _________ |

Abbreviations: AMD: age-related macular degeneration

4. Have you ever been referred from the hospital in Matsu to Taiwan because of eye diseases?

□Yes, please proceed to question 4-1; □No, please skip to question 5

4-1 IF YES: What was the cause of referral? (multiple choice)

□Ophthalmic examination and diagnosis □Ophthalmic laser treatment □Ophthalmic surgery ___________ □For ophthalmic drugs

□Other ___________

**Individual health habits in recent one year**

5. Drinking habit (including various alcoholic beverages, medicated wines but

not wines added in cooking):

| □Never | □Less than once a month | □Once/twice a month |
| --- | --- | --- |
| □Once a week | □Once every 2~3 days | □(Almost) every day |

6. Smoking habit: □Never □Quit, had smoked for ____ years, ____ packs per day

□Yes, for ____ years, ____ packs per day

7. Average daily sun exposure time:

□1 hour or less □1~3 hours □3~5 hours

□5~8 hours □8 hours or more

Please respond the extent of your agreement with each of the following statements:

|  | Strongly agree | Agree | Neutral | disagree | Strongly disagree |
| --- | --- | --- | --- | --- | --- |
| 8. *I should avoid outdoor activities in harsh sunlight* | □ | □ | □ | □ | □ |
| 9. *I should wear a broad-brimmed hat or eyeglasses (including clear prescription lenses, contact lenses, sunglasses) in harsh sunlight* | □ | □ | □ | □ | □ |

Please check the following boxes according to your daily practices:

|  | (Almost)  every day | 3~5 days/week | 1~2 days/week | < 1 day/week | (Almost) never |
| --- | --- | --- | --- | --- | --- |
| 10. *How often do you work or exercise in windy outdoor space?* | □ | □ | □ | □ | □ |
| 11*. How often do you work or exercise beside transparent windows in the daytime?* | □ | □ | □ | □ | □ |
| 12*. How often do you work or exercise in harsh sunlight?* | □ | □ | □ | □ | □ |
|  | (Almost)  every  time | Often | Sometimes | Rarely | Never |
| 12-1*. How often do you wear eyeglasses (including clear prescription lenses, contact lenses, sunglasses) in*  *harsh sunlight?* | □ | □ | □ | □ | □ |
| 12-2*. How often do you wear a broad-brimmed hat or use an umbrella in harsh sunlight?* | □ | □ | □ | □ | □ |

**Demographic Information**

13. Gender: □Male □Female

14. Age: _______ years

15. Current residence: ________village

16. How long have you lived in Matsu?

　 □ <1 year □ 1~5 years □ 6~10 years □ 11~15 years

□ 16~20 years □ 21~30 years □ 31~40years □ 41 years or more

17. Religion:

　 □ None 　 □ Folk religion □ Buddhist □ Taoist

□ I-Kuan Taoist 　 □ Christian □ Catholic □ Muslim

□ Other ____

18. Highest education level:

　 □ Illiterate 　 □ Literate without diploma □ Elementary school

□ Junior high school 　□ Senior high (vocational) school

□ Junior college □ University/college □ Graduate school

□ Other ____

19. Marital status: □ Never married □ Married (or cohabiting)

□ Separated or divorced □ Widowed

20. Current living state:

□ Alone □ With spouse only □ With children

□ With spouse and children (including extended family)

□ With other relatives or friends □ Other ______

21. Main occupation:

□ Homemaker □ Military □ Civil servant □ Teacher

□ Laborer □ Business □ Farming, forestry □ Fishing

□ Livestock farming □ Self-employed □ Student

□ No/unemployed/retired □ Other ____

22. Have you purchased commercial health insurance besides National Health Insurance? □ Yes □ No □ Unknown

23. Annual household income (ten thousand New Taiwan dollars):

□ 30 or less □ 31~50 □ 51~80 □ 81~100 □ 101~120 □ 121~150 □ 151~200 □ 201 or more
