# Supplementary material for: Relationship between practices of eye protection against solar ultraviolet radiation and cataract in a rural area
Source: PLoS One. 2021 Jul 29;16(7):e0255136. doi: 10.1371/journal.pone.0255136 (PMC8321156; doi:10.1371/journal.pone.0255136)
Supplement: S2 File — Chinese version. (DOC) [file pone.0255136.s002.doc]

**個人健康情況問卷**

**1.您是否有下列慢性病(可複選)：**

| □_a_無 | □_b_糖尿病 | □_c_高血壓 | □_d_腦中風 |
| --- | --- | --- | --- |
| □_e_心臟病 | □_f_慢性肺病 | □_g_氣喘 | □_h_肝硬化 |
| □_i_末期腎病(洗腎) | □_j_風濕性關節炎 | □_k_其他疾病___________ | |

2.您平日有無需要戴眼鏡(包括隱形眼鏡)?

□_1_有，請續答第2-1題； □_2_無，請跳答第3題

2-1承上題，請問需要戴眼鏡的原因是什麼?(可複選)

□_1_近視 □_2_遠視 □_3_散光 □_4_老花眼 □_5_不知道 □_6_其他___________

3.您是否曾被醫師診斷下列眼科疾病(可複選)：

| □_a_無  □_c_乾眼症 | □_b_高度近視(任一眼>600度)  □_d_眼翳(翼狀贅片、肉片、肉膜) | | |
| --- | --- | --- | --- |
| □_e_輕微白內障 | □_f_(中度)白內障 | □_g_嚴重白內障 | □_h_青光眼 |
| □_i_角膜病變 | □_j_老年性黃斑部病變 | □_k_糖尿病網膜病變 | □_l_網膜破洞或剝離 |
| □_m_眼球外傷 | □_n_眼瞼皮膚癌 | □_o_砂眼 | □_p_其他__________ |

4.您是否曾經因為眼睛的疾病由馬祖醫療院所轉診到台灣就診?

　 □_1_是，請續答4-1小題； 　　　 □_2_否，請跳答第5題

4-1.承上題，請問轉診的原因是什麼?(可複選)

| □_1_眼科檢查診斷 | □_2_眼科雷射治療 | □_3_眼科手術____ | □_4_拿眼藥 |
| --- | --- | --- | --- |
| □_5_其他__________ |  |  |  |

**以下請教您最近這一年個人健康生活習慣**

5.喝酒習慣(包括各種酒類、保力達B、維士比、與藥酒，但不包括煮菜時加進去的酒)：

| □_1_沒有(滴酒不沾) | □_2_每月喝不到一次 | □_3_每月喝一、二次 |
| --- | --- | --- |
| □_4_每週喝一次 | □_5_兩、三天喝一次 | □_6_(幾乎)每天喝 |

6.吸菸習慣：□_1_沒有 □_2_已戒菸，曾吸菸_______a年，一天_______b包

□_3_有吸菸，已吸菸_______c年，一天_______d包

7.平均每天日曬時間：□_a_1小時內 □_b_1~3小時內 □_c_3~5小時內

□_d_5~8小時內 □_e_8小時以上

請回答您對下列敘述同意的程度:

|  | 非常同意 | 同意 | 無意見 | 不同意 | 非常  不同意 |
| --- | --- | --- | --- | --- | --- |
| 8.強烈陽光下應盡量避免外出 | □_1_ | □_2_ | □_3_ | □_4_ | □_5_ |
| 9.強烈陽光下外出時應戴帽或戴眼鏡(含一般眼鏡、隱形眼鏡或太陽眼鏡) | □_1_ | □_2_ | □_3_ | □_4_ | □_5_ |

以下請按照您的日常活動勾選適當的答案:

|  | 幾乎  每天 | 每週3~5天 | 每週1~2天 | 每週不到1天 | 幾乎沒有 |
| --- | --- | --- | --- | --- | --- |
| 10.您常在風大的戶外活動或工作嗎? | □_1_ | □_2_ | □_3_ | □_4_ | □_5_ |
| 11.您白天常在透光的窗戶旁邊活動或工作嗎? | □_1_ | □_2_ | □_3_ | □_4_ | □_5_ |
| 12.您常在強烈陽光下外出活動或工作嗎? | □_1_ | □_2_ | □_3_ | □_4_ | □_5_ |
|  | 幾乎  每次 | 經常 | 有時候 | 很少 | 沒有 |
| 12-1.強烈陽光下外出時，您常戴眼鏡(含一般眼鏡、隱形眼鏡或太陽眼鏡)嗎? | □_1_ | □_2_ | □_3_ | □_4_ | □_5_ |
| 12-2.強烈陽光下外出時，您常戴遮陽帽或撐傘嗎? | □_1_ | □_2_ | □_3_ | □_4_ | □_5_ |

**基本資料**

13.性別：□_1_男 □_2_女

14.年齡： _________ 歲(實歲)

15.目前居住地點：________村

16.在馬祖居住多久?

　　□_a_1年以下 □_b_1～5年 □_c_6～10年 □_d_11～15年 □_e_16～20年

　　□_f_21～30年 □_g_31～40年 □_h_41年以上

17.宗教信仰：

　 □_1_沒有任何宗教信仰 □_2_燒香、拜拜或是民間傳統信仰 □_3_佛教 □_4_道教 □_5_一貫道 □_6_基督教 □_7_天主教 □_8_回教 □_9_其他___________

18.最高學歷：

　 □_1_不識字 　□_2_識字但未有學歷 □_3_小學 □_4_國(初)中 　□_5_高中(職)

　□_6_專科(含二、三、五專) 　□_7_大學 □_8_研究所及以上 □_9_其他______

19.婚姻狀況： □_1_從未結婚 □_2_已婚(含同居) □_3_分居或離婚 □_4_喪偶

20.目前的居住狀況：

□_1_獨居 □_2_與配偶兩人同住 □_3_與子女同住 □_4_與配偶及子女同住(含三代同堂) □_5_與其他親友同住 □_6_其他____________

21.主要職業：

□_a_家管 □_b_軍 □_c_公務人員 □_d_教 □_e_勞工 □_f_商 □_g_農林 □_h_漁

□_i_畜牧 □_j_自由業 □_k_學生 □_l_無業/失業/退休 □_m_其他______

22.(除全民健保外)您有否購買另外的商業醫療保險：□_1_有 □_2_沒有 □_3_不知道

23.家庭年收入(萬)：□_a_30以下 □_b_31~50 □_c_51~80 □_d_81~100 □_e_101~120 □_f_121~150 □_g_151~200 □_h_201以上
